# Editorial: Insights in General Pediatrics and Pediatric Emergency Care: 2021

**DOI:** 10.3389/fped.2022.981185

**Published:** 2022-07-19

**Authors:** Jérémie F. Cohen

**Affiliations:** Department of General Pediatrics and Pediatric Infectious Diseases, Université Paris Cité, APHP, Necker-Enfants Malades Hospital, Paris, France

**Keywords:** General Pediatrics, Pediatric Emergency Care, fever, social determinants in health, adolescent health, cardiopulmonary arrest

## Background

The scope of the Research Topic “*Insights in General Pediatrics and Pediatric Emergency Care 2021*” was broad, and we received 16 manuscripts from different parts of the globe. Only four papers were accepted for publication, reflecting our current policy in the General Pediatrics and Pediatric Emergency Care section to be more selective. Published manuscripts covered important topics such as fever management, cardiopulmonary arrest, social determinants of health, and adolescent use of emergency medicine services. These topics received priority because of their relevance to our broad audience of pediatricians both in hospital- and office-based practice.

## Summary of published studies

In a first manuscript, Chiappini et al. conducted a Delphi exercise to assess the level of consensus among pediatricians regarding the management of fever. Fever is highly frequent and yet often a source of concern because parents and physicians fear bacterial infections that can sometimes be severe. The study was motivated by the publication of national guidelines in 2017 and also incorporated considerations about the management of fever in telemedicine services, which were massively deployed during the COVID-19 pandemic. The Delphi study consisted of two rounds of online and anonymous questionnaires sent to a panel of 500 pediatricians from different Italian regions, mostly working in private practice (72%). The response rate was only 16%, but such rates are common in online surveys. Consensus was defined as a majority vote of at least 75% for an item. The questionnaires had different topics, including the management of fever in children with COVID-19 and the remote management of the febrile child (i.e., telemedicine). There was consensus on all statements, which aligned with current recommendations, except for one regarding the use of ibuprofen in children with lower respiratory tract infections. Italian pediatricians seem ready to follow current clinical practice guidelines, but further studies should investigate whether these findings from expert professionals are a good reflection of actual clinical practices in the outpatient setting.

In another manuscript, Lawson et al. from Houston, USA, used a retrospective design to investigate the association between social determinants of health and health outcomes in the inpatient setting. This is important since social determinants of health may impact all facets of clinical care. The study relied on data from more than 3,000 unique patients in a large academic pediatric hospital in the US. The studied social determinants included a broad array of patient characteristics such as gender, ethnicity, language, income, and insurance status. Outcomes included rapid response utilization, hospital length of stay, transfer to the intensive care unit, critical deterioration, and mortality. The authors found significant variability in health outcomes according to social characteristics. For example, families who were non-English speaking were less likely to activate the rapid response system, length of stay was longest for patients speaking languages other than Spanish or English, critical deterioration was more common in patients with government insurance, and Hispanic patients had higher odds of mortality. These results highlight the existence of disparities in the management of pediatric inpatients according to their social characteristics in the USA, and the need for further studies and specific interventions to improve health equity.

In a brief research report, Cozzi et al. described adolescents' use of pediatric emergency services in an Italian tertiary care facility in 2018, i.e., before the COVID-19 pandemic. The authors retrospectively analyzed the medical records of adolescents who accessed the emergency department and described causes of access, final diagnoses, and admission rates. In the 3,895 unscheduled adolescent visits, the principal reasons for access were trauma (45%) and organic diseases (39%), and admission rates were low (4%). In this study, mental health problems represented a small proportion of emergency department visits (6%) but were the leading cause of urgent admissions (38%). This is critical since the COVID-19 pandemic and its control measures seem associated with a surge in mental health problems in children and adolescents, which could cause a wave of unexpected admissions. A concomitant decrease in available pediatric beds due to medical and paramedical staff burnout could cause a crisis in several hospitals. A follow-up study would be relevant, as well as other studies in different countries and settings.

Finally, Ding et al. aimed to investigate the epidemiology of cardiopulmonary arrest and outcomes of resuscitation in intensive care units in China. In this large prospective multicenter study conducted in 11 pediatric intensive care units, the authors could include 372 episodes of cardiopulmonary arrests that occurred over 1 year. The main outcome measures included survival and neurological outcomes in survivors. The most frequent initial dysrhythmia was bradycardia (79%). Only 91 children were viable at discharge, and among them, less than half (47%) received a good neurological score. This large multicenter prospective study conducted in China depicts a grim situation where survival and long-term prognosis after pediatric cardiopulmonary arrest appeared relatively poor, which should prompt concern and corrective actions.

## Commentary

The Research Topic “*Insights in General Pediatrics and Pediatric Emergency Care 2021*” had a broad scope and attracted several manuscripts of high quality, including four of which were eventually published. The selected manuscripts covered key aspects of pediatric clinical care, including the management of fever, pediatric emergency departments use by adolescents and the growing concern regarding mental health problems in this specific age group, inpatient care and its social determinants, and cardiopulmonary arrest in the intensive care setting. These studies remind us that children are a fragile population with special needs because of their high dependence on their caregivers and environment and because they are individuals in development. Despite the recent advancements and promises of modern medicine, sources of concern for pediatricians remain the same and deserve further observational and interventional research. Our new Research Topic “*Insights in General Pediatrics and Pediatric Emergency Care 2022*” is now open for submissions.

## Author contributions

The author confirms being the sole contributor of this work and has approved it for publication.

## Conflict of interest

The author declares that the research was conducted in the absence of any commercial or financial relationships that could be construed as a potential conflict of interest.

## Publisher's note

All claims expressed in this article are solely those of the authors and do not necessarily represent those of their affiliated organizations, or those of the publisher, the editors and the reviewers. Any product that may be evaluated in this article, or claim that may be made by its manufacturer, is not guaranteed or endorsed by the publisher.

